# Growth of microorganisms in an interfacially driven space bioreactor analog

**DOI:** 10.1038/s41526-020-0101-4

**Published:** 2020-04-08

**Authors:** Joe A. Adam, Shreyash Gulati, Amir H. Hirsa, Richard P. Bonocora

**Affiliations:** 1 Department of Mechanical, Aerospace and Nuclear Engineering, Rensselaer Polytechnic Institute, Troy, NY 12180-3590 USA; 2 Department of Chemical and Biological Engineering, Rensselaer Polytechnic Institute, Troy, NY 12180-3590 USA; 3 Department of Biological Sciences, Rensselaer Polytechnic Institute, Troy, NY 12180-3590 USA

**Keywords:** Microbiology, Aerospace engineering, Fluid dynamics

## Abstract

Fluid bioreactors in microgravity environments may utilize alternative methods of containment and mixing. The ring-sheared drop (RSD) is a containerless mixing device which functions in microgravity using surface tension for containment and mixes through interfacially-driven flow. To assess the feasibility of using interfacially driven flow devices, such as the RSD, as bioreactors, *Escherichia coli* growth and recombinant protein expression were analyzed in a ground-based analog of the RSD called the knife edge surface viscometer (KEV). Results demonstrated that the KEV can facilitate the growth of *E. coli* and that growth rate increases logarithmically with increasing knife edge rotation rate, similar to the standard growth method on Earth (orbital shaker). Furthermore, the KEV was shown to be viable for supporting recombinant protein expression in *E. coli* at levels comparable to those achieved using standard growth methods.

## Introduction

As space exploration becomes more prevalent, reaches further, and missions last longer, production of the amenities that are enjoyed on Earth will be necessary for long-term success. One terrestrial-based amenity is the use of microorganisms to produce a number of goods and services (such as bioremediation and waste management, large-scale bioreactors, and the production of recombinant proteins and pharmaceuticals). Availability of pharmaceuticals remains a challenge for modern space exploration^1,2^. Controlled growth of microorganisms on Earth has been developed and used effectively for decades, however, the equipment (orbital shakers (OSs), fermenters, etc…) are not compatible with spaceflight. For example, the OSs that are standard bioreactors for growing liquid cultures at liter scale, produce accelerations that may not be suitable for operation on a spacecraft. Thus, there has yet to be a viable bioreactor for large-scale growth of microorganisms in microgravity^3,4^. A bioreactor for microgravity has the potential to support human health in space exploration and may also enable the so-called low Earth orbit economy.

The major obstacle for a bioreactor in microgravity is gas exchange occurring at interfaces of the fluid system. Surfaces and interfacial hydrodynamics within bioreactors are important considerations in the growth of microorganisms^5^. Systems without free surfaces such as the dynamic cell culturing system and space bioreactors I and II (SBR I and SBR II) are limited to small volumes of only a few milliliters^3^. In many space-based bioreactor designs gas exchange occurs via membranes, such as in the clinostat and NASA’s rotating wall vessel^3,6^, or by transparent-walls as in gas-lift photobioreactors present in ESA’s MELISSA^7,8^. However, membranes are less efficient than free surfaces and their effectiveness for microgravity oxygen transport remains undetermined^9^. In addition, biofouling is a major concern, reducing membrane nutrient flux by as much as a third^10^ and light transmittance by over half^11^. In addition to the physical challenges of space bioreactors, microorganisms exhibit altered behavior in microgravity^4,12–14^. As such, specialized microgravity bioreactors distinct from current space or ground-based culturing methods must be designed in order to support biological systems in microgravity.

**Fig. 1:**
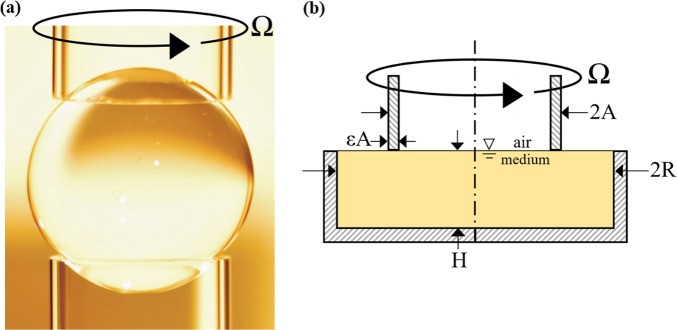
Schematic of the ring-sheared drop (RSD) and its ground-based analog, the knife edge surface viscometer (KEV). **a** Photograph of an oil-water RSD under static conditions (Ω = 0). **b** Schematic of the KEV denoting the knife edge radius A, cylinder radius R, knife edge angular velocity Ω, cylinder height H, and knife edge thickness εA. Computational results of fluid flow in these geometries can be found in Supplementary Fig. 2.

A potential solution for space-based bioreactors comes in the form of containerless liquid media systems that provide sufficient mixing and abundant opportunity for gas exchange. One such system, the ring-sheared drop (RSD, Figs 1a and S1) is a space-specific hardware inspired by the possibility of microgravity experiments with centimeter-scale, and larger, liquid systems without a container, where surface tension provides containment of the liquid. A liquid drop is constrained between two rings in the RSD. One of the rings rotates while the other is held stationary. This differential rotation produces shearing of the interface which in turn drives flow and mixing in the bulk through the action of surface shear viscosity^15^. The RSD technology was developed to study the formation of protein amyloid fibrils, which play a central role in many neurodegenerative disorders including Alzheimer’s disease and Parkinson’s^16^. The RSD offers several fundamental advantages over previous concepts for spaced-based bioreactors: (i) readily scalable—functions with volumes <1 mL to about 1000 mL, (ii) no barriers to gas exchange—virtually the entire surface is free, (iii) no barriers to light transmission—by virtue of not having solid walls, there is no need for optical windows that can be fouled, (iv) energy efficient mixing—due to the fact that mixing in the bulk is conveyed by surface shear viscosity, and finally (v) low reactor weight—since there is no solid container^15,17^.

Because the RSD requires microgravity, an analog system, the knife edge surface viscometer (KEV, Fig. 1b)^18^ was utilized for the present ground-based study. Similar to the RSD, mixing in the KEV is conveyed through the action of surface shear viscosity, resulting in secondary flow effects (Fig. S2). However, unlike the RSD, containment in the KEV is achieved using a glass dish, not by surface tension. Many aspects of KEV hydrodynamics have been investigated recently through computations and experiments, including the dynamics of interfacial flow and bulk flow coupling, and the effects of non-Newtonian interfaces^18,19^. However, previous work has not explored the use of the KEV as a bioreactor or the importance of interfacial hydrodynamics as applied to the growth of microorganisms.

*Escherichia coli*, a Gram-negative, facultative anaerobic bacterium, is the primary bacterium used in synthetic biology to produce proteins and small molecules for both research and clinical applications on Earth. It is possibly the best studied organism with a vast wealth of information in the literature spanning decades of research and thus makes an ideal model system. From a fluid dynamics perspective, shear flows have been shown to affect the behavior of *E. coli*^20–22^, especially with respect to biofilm formation^23–25^. In many scenarios, *E. coli* also interacts with liquid-solid^26–29^ and liquid-air interfaces^30–33^. While *E. coli* is able to grow anaerobically, increased gas transport by turbulent flow has been shown to increase the growth rate of *E. coli* cultures^34^.

The present study uses the KEV and *E. coli* to investigate the role of shear and interfacial hydrodynamics on the growth and recombinant protein expression of *E. coli*. Results support the use of the RSD as a space-based bioreactor.

## Results

### Comparison of *E. coli* growth in the knife edge surface viscometer and orbital shaker

**Fig. 2:**
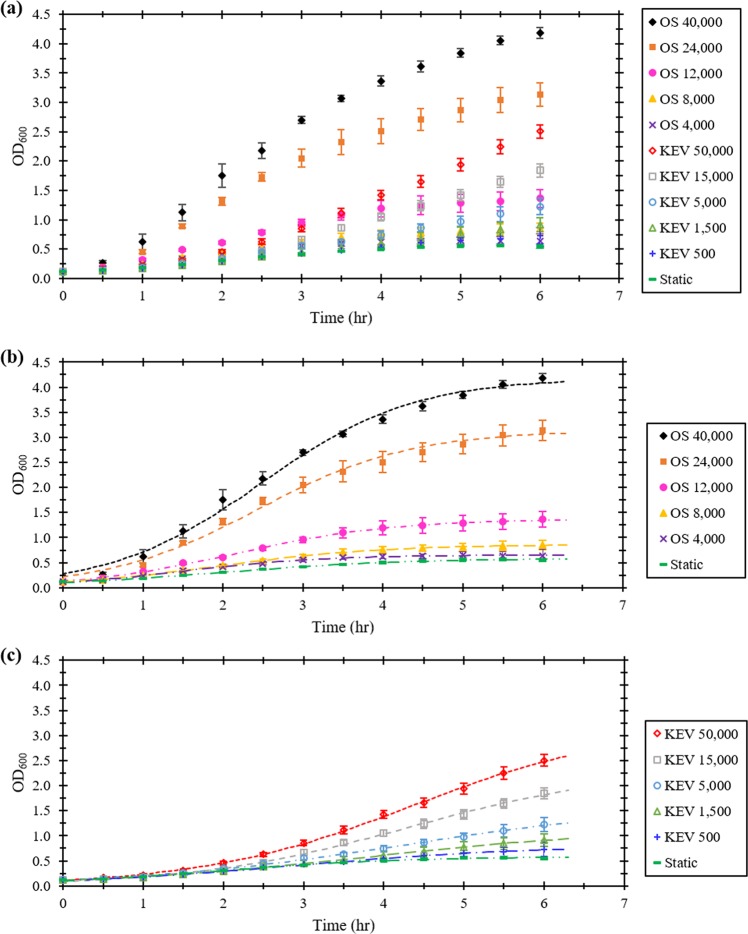
Growth of *E. coli* in the knife edge viscometer compared with growth in a standard orbital shaker at different speeds, nondimensionalized as Reynolds numbers. **a** Optical density at 600 nm plotted as a function of time for growth in the orbital shaker and knife edge viscometer. **b** Growth data for orbital shaker cases. **c** Growth data for knife edge viscometer cases. Lines in (**b**) and (**c**) represent three-parameter logistic equation fits. Error bars represent 95% confidence intervals.

Aerobic growth of *E. coli* was measured in two geometries: a standard OS and the KEV. A control hydrostatic case (“static case”), with no forced flow was performed alongside each experimental case. Reynolds number (Re), was utilized to nondimensionalize rotation rate in each of the geometries used (see “Methods”). A series of controls were performed by growing *E. coli* in the OS over one decade of bed rotation rates ranging from 250 rpm down to 25 rpm, which correspond to Re of 40,000 to 4000, respectively. KEV cases were performed over two decades of Re from 50,000 down to 500 to assess microbial growth in an interfacially driven bioreactor (Fig. 2a). The lower range of Re was based on the minimum speed required to produce a signal. The maximum Re was limited by surface instabilities, requiring a speed below that of spray formation. Re ranges over two decades, spanning from axisymmetric laminar flow to unstable turbulent flow regimes.

Microbial growth curves were quantified using an analytical solution of the logistic equation (see Supplementary Material). Standard cultivation devices such as the OS produce sigmoidal growth curves of this form^35^. To assess the properties of each experimental case, a three-parameter logistic equation model was fitted to each of the growth curves presented in Fig. 2a. Data with these fits is presented separately for OS (Fig. 2b) and KEV (Fig. 2c) trials.

Growth was best using the OS at a Re value of 40,000 (250 rpm), which is a standard *E. coli* growth condition. The fastest KEV case (KEV 50,000) produced a final OD_600_ about 60% of this standard laboratory growth method. Significant growth above the static culture was observed at *Re* = 8000 and above (Fig. 2b) for the OS cultures, and Re = 5000 and above for KEV cultures (Fig. 2c). For OS and KEV cultures, the same saturation value was reached after ~48 h of growth, corresponding to an OD_600_ of 4.96 ± 0.29 (95% CI). Inspecting high shear growth curves from each of the two apparatuses (OS 40,000, OS 24,000, KEV 50,000, and KEV 15,000), the slopes of KEV growth curves are larger than OS slopes at the end of the experiment indicating that the high Re KEV trials may still be in the exponential phase.

**Fig. 3:**
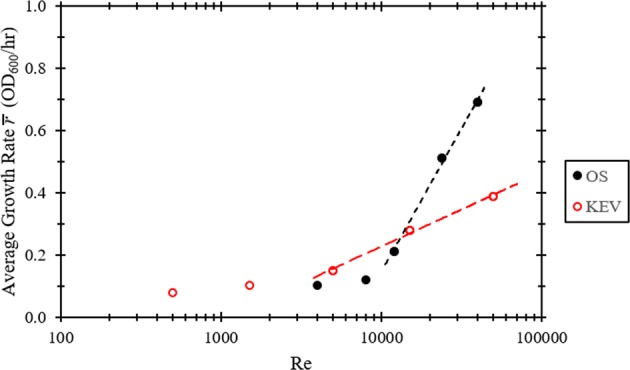
Average growth rates for each of the orbital shaker and knife edge viscometer cases as a function of Reynolds number. Average growth rate is defined according to Eq. 6 in the Supplementary Information. Lines represent logarithmic functions fitted to the last three data points of each curve.

Each fit from the results presented in Fig. 2 was comprised of three parameters: *K*, saturation level, *y*_0_, initial population value, and *r*, intrinsic growth rate. These parameters were used to calculate an average growth rate for each experimental case. Average growth rate, $\bar r$, is defined in terms of the three model parameters and the number of hours within the sampling period, Δt (see Supplementary Information). Average growth rates, $\bar r$, for each case plotted against Re are presented in Fig. 3.

Logarithmic fits in Fig. 3 show that Re at and above 5000 for the KEV and 12,000 for the OS, result in logarithmically increasing average growth rates. The static case exhibits the lowest average growth rate among all cases at 0.07 OD_600_/h, reaching an endpoint growth of ~13% of the standard OS 250 growth case. Growth rate increases more quickly for the OS geometry, likely a consequence of substantial surface waves in the OS that result in significantly enhanced gas exchange^36^. Furthermore, logarithmic fits intersect one another at a Re of ~13,000, indicating that at this point conditions in the two geometries result in about the same average growth rate. High Re flow regimes likely produce greater availability to nutrients due to increased secondary flow and the manifestation of instabilities and ultimately turbulence which produce significantly enhanced mixing^37^. At Re up to the order of 10^3^ the flow in the KEV is stable, remaining axisymmetric and steady^38^.

**Fig. 4:**
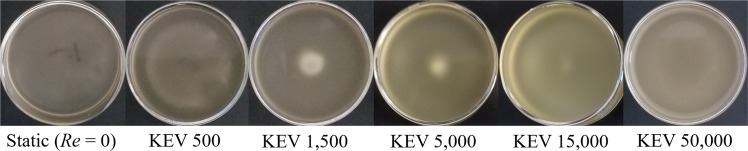
Photographs of *E. coli* floor patterns in the knife edge viscometer dishes after 6 h of growth. The five experimental cases at different Reynolds numbers are displayed along with the static case (Re = 0).

The effect of secondary flow on the distribution of cells throughout the dish in the KEV cultures at the end of each experiment was documented by imaging the pattern on the floor (Fig. 4). Endpoint floor pattern images occurred after 6 h of growth and display dishes that are no longer being sheared. Flask floors used in the OS were not recorded as no patterns were evident. Floor pattern photographs of the KEV dishes presented in Fig. 4 show circular patches of cells gathered on the bottom of each dish. No patch was observed in the static case, with the floor of the dish covered in an essentially uniform distribution of cells. As Re increases, patterns begin to form, such that cells collect in the center of the dish. These patch radii decrease with increasing Re to Re = 5000. At Re values above 5000 the patch radii become larger and less distinct and the density of cells across the floor is more diffuse, most likely due to increased turbulence.

### GFP expression in the KEV and OS

**Fig. 5:**
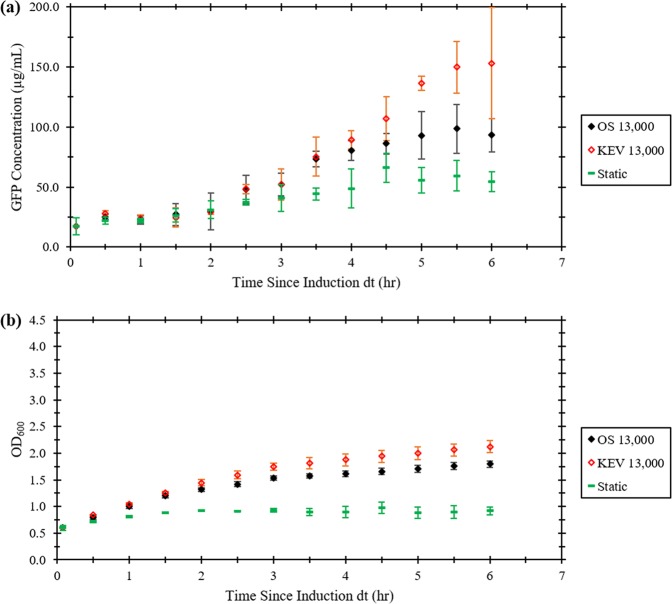
Growth and protein expression in the orbital shaker and knife edge viscometer at the equal average growth rate Reynolds number of 13,000. The static case is included as a baseline control. **a** GFP concentration in µg/mL versus time since induction. **b** OD_600_ versus time since induction. Error bars represent 95% confidence intervals.

One of the end goals of using an interfacially sheared system to grow microorganisms is for the production of protein-based pharmaceuticals. Growth and expression of the recombinant reporter protein, green fluorescent protein (GFP) was compared in the OS and KEV at similar average growth rates and to a static control. Protein expression was compared in the OS and KEV at a Re value of 13,000. This value was determined by the intersection between the fitted logarithmic functions in Fig. 3 to produce similar average growth rate, $\bar r$, in each system. Batch cultures were grown at 250 rpm to an OD_600_ of 0.40, induced with 0.02% arabinose, and split into separate OS, KEV and static cultures were monitored for GFP fluorescence every 30 min for 6 h (Fig. 5a). OD_600_ readings were also recorded (Fig. 5b). Both the OS and KEV cultures grew significantly better than the static control and growth in the KEV is slightly better than the OS culture (Fig. 5b). GFP production was roughly the same for each culture for the first three hours. After this point, GFP production was significantly higher in the KEV and OS cultures as compared with the static culture.

## Discussion

Decades of research have settled on an optimal system for the controlled growth of bacteria and the expression of recombinant genes/proteins in Earth-based systems, most notably growth of *E. coli* with vigorous agitation in OSs. As the need arises to perform these same functions in the microgravity environment of space, the usefulness of Earth-optimized hardware is limited to obsolete. Development of new growth systems that take advantage of microgravity are necessary. To this end the RSD has been developed to provide sufficient containment of fluids, without the need for solid structures. Mixing of fluids is performed by surface shear viscosity.

In the present study, the ability of surface shear viscosity to support adequate mixing was investigated using the KEV, a ground-based analog of the RSD. Comparison of the *E. coli* growth in the KEV to a traditional ground-based OS system showed that the fastest KEV case (KEV Re 50,000) produced a final OD_600_ about 60% of the standard laboratory growth method (OS at 250 rpm, corresponding to a Re of 40,000; Fig. 2a). Observing growth data in Fig. 2a, larger Re leads to greater overall growth in both the OS and KEV. This result is quantified by the monotonic increases in average growth rate in Fig. 3. The shape of the logistic equation fits in Fig. 2b, c can be used to examine the relationship between the phases of bacterial growth and Re. These experimental curves have similar shape, including initial lag phases, an exponential phase, and a stationary phase, all of which depend on Re. Time spent in the lag phase decreases with increasing Re due to improved mixing, most likely because of increased access to nutrients and aeration. Similarly, growth rate and final OD_600_ of the exponential phase, both increased with Re due to mixing (Fig. 2).

Floor pattern photographs of the KEV dishes presented in Fig. 4 show circular patches of cells gathered on the bottom of each dish. No patch was observed in the static case, with the floor of the dish covered in an essentially uniform distribution of cells. As Re increases, patterns begin to form due to secondary flow in the KEV caused by inertia flinging fluid at the interface away from the knife edge^18^. This surface flow is then directed downward by the cylinder wall, and subsequently, turned inward by the floor that forces cells toward the center of the dish (Fig. S2). In the cases where the flow is expected to be steady (KEV 500 and KEV 1500), patch radius decreases as Re increases. In the KEV 500 case, patch radius is very large, with cells concentrated toward the periphery of the patch. At higher Re of 5000 and larger, the flow likely becomes unsteady^18^. In these unsteady regimes floor patterns become diffuse due to instability^39^. The effects of turbulent dispersion are most evident in the final KEV 50,000 case, where the magnitude of velocity fluctuations has completely dispersed the central patch. This could have important implications for RSD function in microgravity. Since there are no container walls or gravity to push and pull cells in a specific direction a similar collection of cells may occur at the poles of the drop, especially the pole opposite the rotating ring. Experiments in microgravity are necessary to evaluate this phenomenon.

Note, that the sampling method used to measure growth and protein expression for the KEV was devised to be minimally disruptive to fluid flow, taking samples near the surface of cultures. This sampling method likely drew from regions which contained fewer cells when compared with media closer to the floor of the dish. This is because cells and nutrients may sediment or be driven to the floor via secondary flow, as demonstrated by the floor patterns in Fig. 4. Therefore, growth and protein expression measurements in this investigation likely underestimate levels that could be achieved, and represent lower limits of these values. Endpoint OD_600_ and GFP measurements where the floor was mixed to assess the effect of sedimentation confirm slight underestimation of these values in the KEV and OS within 5% of standard endpoint measurements.

As predicted (Fig. 3), growth at a Re value of 13,000 is nearly identical in the KEV and OS geometries, with a slightly higher rate with the KEV (Fig. 5b). Protein production is comparable in the KEV and OS (Fig. 5a). This suggests that at certain growth rates, surface shear is as effective as shaking for protein expression. Furthermore, in both geometries cell growth is significantly better and produces more protein than the static culture (Fig. 5). This signifies that surface shear is a viable means of mixing in bioreactors for recombinant protein production in microorganisms.

In summary, the KEV appears capable of facilitating the growth of *E. coli* to within 60% of standard culturing methods in an equal period of time. Growth rate increases with increasing Re, likely due to enhanced mixing through secondary flow and ultimately turbulence. At low Re, the KEV performs on par with the OS. Furthermore, the KEV was shown to be viable for supporting recombinant protein expression in microorganisms. These results strongly support that interfacially driven flow is a viable means of mixing and promoting proper aeration for the growth of microorganisms and will be crucial for the development of space-based devices such as the RSD as viable bioreactors in the microgravity environment. Further development is necessary to ensure that the RSD can be scaled to a size suitable for manufacturing. Aside from scaling, which is limited by physical factors, reaching production scale will likely require parallelization, such as the use of RSD arrays.

## Methods

### Strains, plasmids, and media

*E. coli* strain BL21^40^ (*E. coli* B F^–^ *ompT gal dcm lon hsdS*_*B*_(*r*_*B*_^–^*m*_*B*_^–^)[*malB*^+^]_K-12_(λ^S^)) was transformed with the plasmid pGLO^41,42^. pGLO contains the GFP gene under control of the arabinose-inducible promoter pBAD^43^, along with an ampicillin resistance gene. LB broth (10 g/L tryptone; 5 g/L yeast extract; 10 g/L NaCl) was supplemented with 100 µg/mL of ampicillin to select for plasmid retention.

### Growth conditions

For the determination of bacterial growth curves, cultures were grown to saturation overnight in LB + ampicillin broth for an average growth time of 20 h. The overnight cultures were diluted in fresh LB medium to obtain 36.5 mL sub-cultures at a starting optical density of OD_600_ 0.10 for OS, KEV, and static conditions. The static condition was an undisturbed dish with the same dimensions used with the KEV, but no added shear or shaking. In this context, “static” refers to the hydrostatic scenario in fluid mechanics. OD_600_ was monitored every 30 min over a period of 6 hr. A total of six replicates were performed for each growth case in the OS and KEV. Thirty replicates were performed for the static case, as this case served as a control during each experiment. At the end of each KEV growth experiment, a photograph of each dish was captured to survey trends in floor patterning.

### Measurement of GFP fluorescence

Protein expression was measured using fluorescence of the reporter gene GFP from the pGLO plasmid. Cultures were grown to saturation overnight in the OS and sub-cultured to OD_600_ 0.10. Sub-cultures were grown in the OS at 250 rpm to an OD_600_ of ~0.40. The sub-cultures were induced by the addition of arabinose to a final concentration of 0.02% by volume to activate GFP expression. Induced cultures were split and grown in both the OS and KEV at the equal growth rate Re of 13,000 and a static case was performed as a baseline control. OD_600_ and fluorescence measurements of 100 µL culture samples were taken every 30 min after induction for a total of 6 h post induction. To maintain a constant volume and contact with the knife edge, samples were replaced in the original cultures. GFP fluorescence was measured using a fluorometer exciting samples at 390 nm and measuring emission at 510 nm. Fluorometer measurements were compared with a standard curve using purified GFP allowing for calculation of GFP mass. Protein expression experiments were conducted in biological triplicate.

### Growth vessel geometries and equipment

A triple KEV array was constructed with three rigid plastic knife edges (made from phenolic resin, radius A = 2.12 cm, and knife edge thickness εA = 0.20 cm) centered over three glass dishes (radius R = 2.85 cm, and height H = 1.40 cm). Rotation rates were set by a stepper motor to achieve the desired values of Re. OS experiments used a Thermo Scientific Model SHKE8000 Series MaxQ OS. Standard 125 mL Erlenmeyer flasks were used in the OS.

### Reynolds number calculation

Equation 1 presents the expression for Re in the KEV in terms of parameters defined in Fig. 1, where the angular velocity Ω is measured in rad/s^18^. Equation (2) defines Re in the OS in terms of orbital bed angular velocity Ω_*O*_ rad/s, and *D* (=6.46 cm), the inner diameter at the base of a 125 mL Erlenmeyer flask. This definition of Re for an Erlenmeyer flask is similar to those defined in other studies^44,45^. For the OS, Re values of 40,000, 24,000, 12,000, 8000, and 4000 correspond to 250, 125, 75, 50, and 25 rpm, respectively. In the calculation of Re, the value of kinematic viscosity, *ν*, was kept constant and assumed to be that of water at 37 °C, the temperature at which all experiments were conducted. The viscosity of water is smaller than that of the *E. coli* culture, leading to over estimation of Re.

1 $${\mathrm{Re}}_{\mathrm{KEV}} \equiv \frac{{{\mathrm{\Omega }}A^2}}{\nu }$$

2 $${\mathrm{Re}}_{\mathrm{OS}} \equiv \frac{{{\mathrm{\Omega }}_O\left( {\frac{D}{2}} \right)^2}}{\nu }$$

### Reporting summary

Further information on research design is available in the Nature Research Reporting Summary linked to this article.

## Supplementary information

Reporting Summary

Supplementary Information

## Data Availability

All data from this investigation, including calibration curves and MATLAB code, is available upon request to the corresponding author.

## References

[1] Blue RS (2019). Supplying a pharmacy for NASA exploration spaceflight: challenges and current understanding. NPJ Microgravity.

[2] Menezes AA, Cumbers J, Hogan JA, Arkin AP (2015). Towards synthetic biological approaches to resource utilization on space missions. J. R. Soc. Interface.

[3] Walther I (2002). Space bioreactors and their applications. Adv. Space Biol. Med..

[4] Horneck G, Klaus DM, Mancinelli RL (2010). Space microbiology. Microbiol. Mol. Biol. Rev..

[5] Persat A (2015). The mechanical world of bacteria. Cell.

[6] Barzegari A, Saei AA (2012). An update to space biomedical research: tissue engineering in microgravity bioreactors. BioImpacts.

[7] Godia F (2002). MELISSA: a loop of interconnected bioreactors to develop life support in space. J. Biotechnol..

[8] Hendrickx L (2006). Microbial ecology of the closed artificial ecosystem MELiSSA (micro-ecological life support system alternative): reinventing and compartmentalizing the Earth’s food and oxygen regeneration system for long-haul space exploration missions. R. Microbiol..

[9] Clement, G. & Slenzka, K. *Fundamentals of Space Biology*, 340–343 (Springer Microcosm Press, CA, 2006).

[10] Takimoto Y, Hatamoto M, Ishida T, Watari T, Yama T (2018). Fouling development in A/O-MBR under low organic loading condition and identification of key bacteria for biofilm formations. Sci. Rep..

[11] Harris L (2013). Potential impact of biofouling on the photobioreactors of the offshore membrane enclosures for growing algae (OMEGA) system. Bioresour. Technol..

[12] Nickerson CA, Ott CM, Wilson JW, Ramamurthy R, Pierson DL (2004). Microbial responses to microgravity and other low-shear environments. Microbiol. Mol. Biol. Rev..

[13] Leys, N. M. E. J., Hendrickx, L., De Boever, P., Baatout, S. & Mergeay, M. Space flight effects on bacterial physiology. *JBRHA* **18**, 193−199 (2004). 15471227

[14] Clement G. *Fundamentals of Space Medicine* 2nd edn, 55–57 (*Springer Microcosm Press*, CA, 2011).

[15] Gulati S, Riley FP, Lopez JM, Hirsa AH (2018). Mixing within drops via surface shear viscosity. Inter. J. Heat. Mass Transf..

[16] Dobson C (1999). Protein misfolding, evolution and disease. Trends Biochem. Sci..

[17] Gulati S, Raghunandan A, Rasheed F, McBride SA, Hirsa AH (2017). Ring-sheared drop (RSD): Microgravity module for containerless flow studies. Microgravity Sci. Techn..

[18] Lopez JM, Hirsa AH (2015). Coupling of the interfacial and bulk flow in knife-edge viscometers. Phys. Fluids.

[19] Raghunandan A, Hirsa AH, Underhill PT, Lopez JM (2018). Predicting steady shear rheology of condensed-phase monomolecular films at the air-water interface. Phys. Rev. Lett..

[20] Kaya T, Koser H (2012). Direct upstream motility in. Escherichia coli. Biophys. J..

[21] Portela R (2016). Rotational tumbling of *Escherichia coli* aggregates under shear. Phys. Rev. E..

[22] Patrıcio P. et al. Living bacteria rheology: population growth, aggregation patterns and cooperative behaviour under different shear flows. https://arxiv.org/abs/1403.1405v1 (2014). 10.1103/PhysRevE.90.022720 25215771

[23] Schembri M, Kjærgaard K, Klemm P (2003). Global gene expression in Escherichia coli biofilms. Mol. Microbiol..

[24] Besharova O, Suchanek V, Hartmann R, Drescher K, Sourjik V (2016). Diversification of gene expression during formation of static submerged biofilms by Escherichia coli. Front. Microbiol.

[25] Alsharif G (2015). Host attachment and fluid shear are integrated into a mechanical signal regulating virulence in Escherichia coli O157:H7. PNAS.

[26] Whitfield M, Ghose T, Thomas W (2010). Shear-stabilized rolling behavior of E. coli examined with simulations. Biophys. J..

[27] Thomas W, Nilsson L, Forero M, Sokurenko E, Vogel V (2004). Shear-dependent ‘stick-and-roll’ adhesion of type 1 fimbriated Escherichia coli. Mol. Microbiol.

[28] Hill J, Kalkanci O, McMurry J, Koser H (2007). Hydrodynamic surface interactions enable Escherichia coli to seek efficient routes to swim upstream. Phys. Rev. Lett..

[29] Kaya T, Koser H (2009). Characterization of hydrodynamic surface interactions of Escherichia coli cell bodies in shear flow. Phys. Rev. Lett..

[30] Wu C, Lim JY, Fuller GG, Cegelski L (2012). Quantitative analysis of amyloid-integrated biofilms formed by uropathogenic *Escherichia coli* at the air-liquid interface. Biophys. J..

[31] Krol JE (2011). Increased transfer of a multidrug resistance plasmid in *Escherichia coli* biofilms at the air-liquid interface. Appl. Environ. Microbiol..

[32] Sinibaldil G, Iebba V, Chinappi M (2017). Swimming and rafting of *E. coli* microcolonies at air–liquid interfaces. MicrobiologyOpen.

[33] Lemelle L, Palierne J-F, Chatre E, Place C (2010). Counterclockwise circular motion of bacteria swimming at the air-liquid interface. J. Bacteriol..

[34] Al-Homoud A, Hondzo M (2008). Enhanced uptake of dissolved oxygen and glucose by Escherichia coli in a turbulent flow. Appl. Microbiol. Technol..

[35] Pla M-L, Oltra S, Esteban M-D, Andreu S, Palop A (2015). Comparison of primary models to predict microbial growth by the plate count and absorbance methods. BioMed. Res. Int..

[36] Jenkins AD (2007). Interaction of waves, surface currents, and turbulence: the application of surface-following coordinate systems. J. Ocean Univ. China.

[37] Dimotakis PE (2000). The mixing transition in turbulent flows. J. Fluid Mech..

[38] Raghunandan A, Lopez JM, Hirsa AH (2015). Bulk flow driven by a viscous monolayer. J. Fluid Mech..

[39] Panton, R. L. *Incompressible Flow*. 4th edn, 772–776 (John Wiley & Sons Inc, NY, 2013).

[40] Kim S (2017). Genomic and transcriptomic landscape of *Escherichia coli* BL21(DE3). Nucleic Acids Res..

[41] Sambrook J, Fritsch EF, Maniatis T (1989). Molecular Cloning: A Laboratory Manual.

[42] BIO-RAD. *Biotechnology Explorer: pGLO Bacterial Transformation Kit*. (BioRad Tech, 2018).

[43] Guzman L, Belin D, Carson M, Beckwith J (1995). Tight regulation, modulation, and high-level expression by vectors containing the arabinose PBAD promoter. J. Bacteriol..

[44] Buchs J, Maier U, Milbradt C, Zoels B (2000). Power consumption in shaking flasks on rotatory shaking machines: I. Power consumption measurement in unbaffled flasks at low liquid viscosity. Biotechnol. Bioeng..

[45] Peter C, Lotter S, Maier U, Buchs J (2004). Impact of out-of-phase conditions on screening results in shaking flask experiments. Biochem. Eng. J..

