# Supplementary material for: Growth of microorganisms in an interfacially driven space bioreactor analog
Source: NPJ Microgravity. 2020 Apr 8;6:11. doi: 10.1038/s41526-020-0101-4 (PMC7142127; doi:10.1038/s41526-020-0101-4)
Supplement: Supplementary file 2 — Supplementary Information [file 41526_2020_101_MOESM2_ESM.pdf]

Supplementary information for: Growth of microorganisms in an interfacially-driven space bioreactor analog

Joe A. Adam

Department of Mechanical, Aerospace and Nuclear Engineering; also,

Biological Sciences,

Rensselaer Polytechnic Institute, Troy, NY, 12180-3590, USA.

Shreyash Gulati

Department of Mechanical, Aerospace and Nuclear Engineering;

Rensselaer Polytechnic Institute, Troy, NY, 12180-3590, USA.

Amir H. Hirsa

Department of Mechanical, Aerospace and Nuclear Engineering; also,

Chemical and Biological Engineering

Rensselaer Polytechnic Institute, Troy, NY, 12180-3590, USA.

Richard P. Bonocora*

Department of Biological Sciences,

Rensselaer Polytechnic Institute, Troy, NY, 12180-3590, USA.

Corresponding Author Contact Information

bonocr@rpi.edu

Supplementary Notes

Geometry of the Ring-Sheared Drop (RSD)

Supplementary Fig. 1 shows the geometric configuration of the RSD considered here. A liquid drop of radius $R_d = 1.27$ cm is constrained between two rings located at polar angles $\theta = 37°$ and $\theta = 143°$. The top ring rotates with an angular velocity Ω and the bottom ring is stationary. The non-dimensional angular velocity of the rotating ring is expressed by the Reynolds number, $Re_{RSD} \equiv (\Omega {R_d}^2)/\nu$.

Surface Shear Viscosity and Boussinesq Number

Certain constituents of LB medium, as well as *E. coli* and the molecules these cells produce can be surface-active and accumulate at the air-water interface. Aside from generally decreasing surface tension, surface-active materials can give rise to a surface (excess) shear viscosity, μ^s.[1] Thus, surface-active material can significantly alter the transport of mass, momentum, and energy at gas-liquid interfaces.[2]

Boussinesq number represents the ratio of surface shear viscosity, μ^s, and the product of dynamic viscosity of the bulk, μ, and a characteristic length scale. For the RSD, $Bo \equiv \mu^s/(\mu R_d)$ and for the KEV, $Bo \equiv \mu^s/(\mu A)$, where A is the (outer) radius of the knife edge.

Numerical Results for Flow Fields in the RSD and Knife-Edge Viscometer (KEV)

To illustrate the basic flow field in the RSD and KEV, numerical simulations were performed at a Re of 500. The relatively low Re ensures that the axisymmetric simulations predict what should be observed in corresponding experiments, avoiding instabilities such as the onset of rotating waves and ultimately turbulence that would be observed at high Re.[3]

Two different Bo, 0.0001 and 100 where simulated representing the two limits of an essentially inviscid surface and a very high surface shear viscosity, respectively. These limits for Bo were found in a previous numerical study of the RSD.[4] Measurements of surface shear viscosity and its likely dependence on the organisms' life cycle was beyond the scope of this investigation.

Supplemental Fig. 2 shows the non-dimensional azimuthal velocity (tangential motion about the axis, shown by the color map) and secondary flow in the rz-plane (shown by the arrows) for the RSD and KEV. As the surface becomes viscous and Bo is increased from 0.0001 to 100, the arrows become larger depicting a stronger secondary flow and hence, greater mixing of the bulk. This trend is similar in both the RSD and KEV. Note, the thickness of the knife-edge and the rings have already been established to play a secondary role in both geometries (RSD and KEV).[4,5]

Numerical Technique for the KEV

Flow in the bulk of the KEV is governed by the Navier-Stokes equations. These equations were non-dimensoinalized using A as the length scale and using $1/\Omega$ as the time scale:

$$\boldsymbol{\nabla}\cdot\boldsymbol{u} = 0\,, \tag{1}$$

$$\frac{\partial \boldsymbol{u}}{\partial t} + (\boldsymbol{u}\cdot\boldsymbol{\nabla}\boldsymbol{u}) = -\boldsymbol{\nabla}p + \frac{1}{Re}\boldsymbol{\nabla}^2\boldsymbol{u}, \tag{2}$$

where $\boldsymbol{u}$ is non-dimensional velocity vector and p is non-dimensional pressure. Utilizing cylindrical coordinates (r, ϕ, z), where r is the radius, ϕ is the azimuth angle (along the direction of rotation) and z is the axial distance, the velocity is $\boldsymbol{u} = (u, v, w)$.

The boundary conditions are no-slip on walls and symmetry on the centerline. At the knife edge, $v = \Omega r$ and $u = w = 0$. At the interface, $u = w = 0$ and v is given by the solution to the azimuthal interfacial stress balance:

$$\frac{\partial v}{\partial z} = Bo\left(\frac{\partial^2 v}{\partial r^2} + \frac{1}{r}\frac{\partial v}{\partial r} - \frac{v}{r^2}\right). \tag{3}$$

Equation 3 is based on the Boussinesq-Scriven model[1,6,7] which assumes a Newtonian interface. More details of the interfacial model and the process used to derive Eqn. 3 can be found in a previous numerical study of the KEV.[5]

COMSOL finite-element method based software was used to compute the flow in the KEV. Second-order shape functions were used for velocity. The dimensions of the KEV considered in the numerical model are same as those in the experimental setup described in the main article. The

mesh at the interface included a fine distribution of nodes in the radial direction and an axial boundary-layer in the bulk. The bulk mesh consisted of $3{,}163$ quadrilateral elements, with a base mesh-element size of 0.02, and the finest size being approximately 9×10^{-4}. All computations were performed using a steady-state solver.[4]

Numerical Technique for the RSD

Using R_d as the length scale and $1/\Omega$ as the time scale, the flow in the RSD is also governed by the Navier-Stokes equations, Eqn. 1 and 2, with the only difference being that the appropriate Re is used (Re_{RSD}). The numerical method used here is identical to that employed in a previous study of the RSD.[4]

Growth Curve Fits

The shape of the growth curve for microorganisms may be explained using population dynamics theory.[8] Specifically, the logistic equation, a first order non-linear ordinary differential equation, may be used to describe the growth of a population using three parameters. The differential form of the logistic equation[9] is given by:

$$\frac{dy}{dt} = r\left(1 - \frac{y}{K}\right) y\,, \tag{4}$$

where y is the dependent variable associated with the magnitude of the population, here being the number of microorganisms, measured as OD_{600}. The independent variable, *t*, is time. The two parameters which appear in this differential form are the intrinsic growth rate *r* (1/hr), and the saturation level *K* (abs). This differential equation can be solved analytically using the initial condition that y at time 0 is equal to some initial value y_0 ($\equiv y(0)$). The analytical solution of the logistic equation is

$$y = \frac{y_0 K}{y_0 + (K - y_0)e^{-rt}}\,. \tag{5}$$

Using the analytical solution of the logistic equation (Eqn. 5), three-parameter models were generated for each of the eleven experimental cases. In each case, y_0, K, and r parameters were determined using a sequential quadratic programming (SQP) optimization scheme which minimized the difference between model predictions and experimental data. Two constraints were

imposed: the saturation level, K, must be positive, and a nonlinear constraint ensuring that the final data point is less than the saturation level. The nonlinear constraint was imposed to produce a better fit for data at later time points which was vital for analysis of protein expression. The three fit constants for each case and Δt, the number of hours within the growth period, were used to calculate an average growth rate, $\overline{r}$, for each experimental case according to Eqn. 6.

$$\overline{r} = \frac{(K - y_0)r}{\Delta t}. \quad (6)$$

Supplementary References:

1. Edwards, D. A., Brenner, H. & Wasan, D. T. *Interfacial Transport Processes and Rheology*, pages 109-110 (Butterworth–Heinemann, Boston, USA, 1991).
2. Hunt, J.C.R. Turbulent structure and turbulent diffusion near gas-liquid interfaces, in: Brutsaert, W. & Jurka G.H. (Eds.). Gas Transfer at Water Surfaces. *Riedel*, 67–82 (1984).
3. Panton, R. L. *Incompressible Flow 4th ed.*, pages 737-771 (Wiley and Sons, Hoboken, NJ, USA, 2013).
4. Gulati, S., Riley, F. P., Hirsa, A. H. and Lopez, J. M. Flow in a containerless liquid system: Ring-sheared drop with finite surface shear viscosity. *Phys. Rev. Fluids* **4**, 044006 (2019).
5. Lopez, J. M. & Hirsa, A. H. Coupling of the interfacial and bulk flow in knife-edge viscometers. Phys. Fluids **27**, 042102 (2015).
6. Scriven L. E. Dynamics of a fluid interface. Equation of motion for Newtonian surface fluids. *Chem. Eng. Sci.* **12**, 98 (1960).
7. Slattery, J. C. , Sagis, L. & Oh, E.-S. *Interfacial Transport Phenomena.* 2nd Edn, pages 358-361 (*Springer*, NY, USA, 2007).
8. Pla M-L., Oltra S., Esteban M-D., Andreu S. & Palop A. Comparison of primary models to predict microbial growth by the plate count and absorbance methods. BioMed Res. Int. 2015, 365025 (2015).
9. Boyce W. & DiPrima R. Elementary Differential Equations and Boundary Value Problems, 7th Edn, pages 74-81 (John Wiley & Sons Inc, NY, USA, 2003).

Supplementary Figures

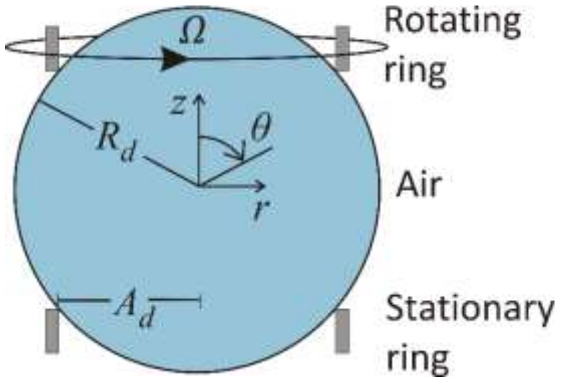

Supplementary Fig. 1 Schematic of the ring-sheared drop (RSD) where R_d is the drop radius, A_d is the ring radius, Ω is the angular velocity of the rotating ring, r is the radial direction in a cylindrical coordinate (r, ϕ, z), z is the axial direction and θ is the polar angle.[4] The rotating and stationary rings are located at polar angles $\theta = 37°$ and $143°$, respectively.

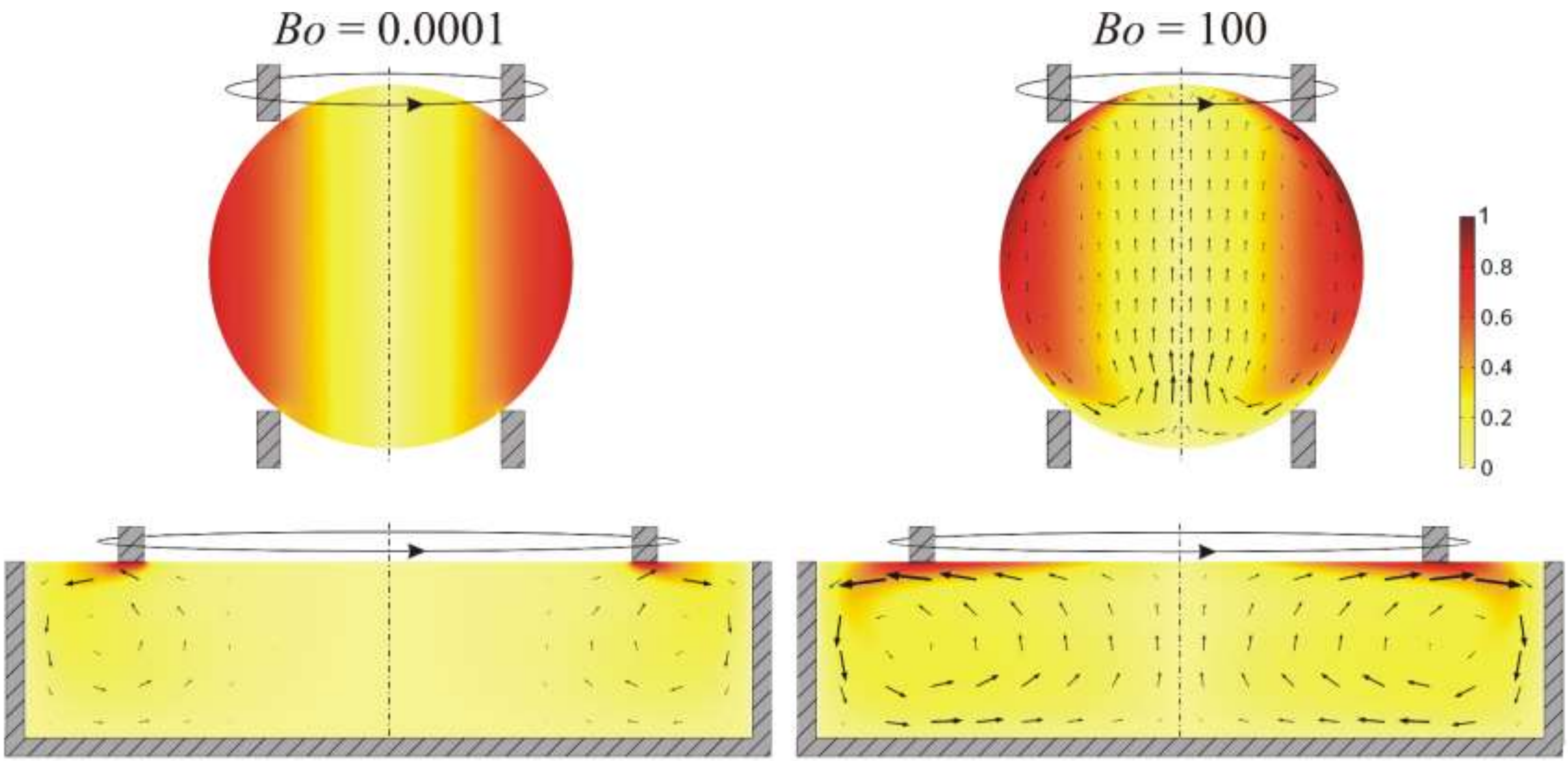

Supplementary Fig. 2 Numerically predicted flow field in the ring-sheared drop (RSD) and knife-edge viscometer (KEV) for the top ring/knife edge rotating at a non-dimensional speed corresponding to a Reynolds number, *Re*, of 500 and non-dimensionalized surface shear viscosities represented by Boussinesq number, *Bo* as indicated. The color map represents non-dimensional azimuthal velocity (tangential motion about the centerline) ranging from yellow (0) to red (1). Vectors represent velocity in the rz-plane (secondary flow).
